# Impact of Implementing a Wiki to Develop Structured Electronic Order Sets on Physicians' Intention to Use Wiki-Based Order Sets

**DOI:** 10.2196/medinform.4852

**Published:** 2016-05-17

**Authors:** Patrick Michel Archambault, Pierre Beaupré, Laura Bégin, Audrey Dupuis, Mario Côté, France Légaré

**Affiliations:** ^1^ Centre intégré de santé et services sociaux de Chaudière-Appalaches Lévis, QC Canada; ^2^ Département de médecine familiale et médecine d'urgence Université Laval Québec, QC Canada; ^3^ Centre de recherche du CHU de Québec Axe Santé des populations - Pratiques optimales en santé, Traumatologie – Urgence – Soins Intensifs Québec, QC Canada; ^4^ Département d'information et de communication Université Laval Québec, QC Canada

**Keywords:** knowledge translation, wiki, collaborative writing applications, decision support tools, health informatics, Theory of Planned Behavior, emergency medicine, computer physician order entry

## Abstract

**Background:**

Wikis have the potential to promote best practices in health systems by sharing order sets with a broad community of stakeholders. However, little is known about the impact of using a wiki on clinicians’ intention to use wiki-based order sets.

**Objective:**

The aims of this study were: (1) to describe the use of a wiki to create structured order sets for a single emergency department; (2) to evaluate whether the use of this wiki changed emergency physicians’ future intention to use wiki-based order sets; and (3) to understand the impact of using the wiki on the behavioral determinants for using wiki-based order sets.

**Methods:**

This was a pre/post-intervention mixed-methods study conducted in one hospital in Lévis, Quebec. The intervention was comprised of receiving access to and being motivated by the department head to use a wiki for 6 months to create electronic order sets designed to be used in a computer physician order entry system. Before and after our intervention, we asked participants to complete a previously validated questionnaire based on the Theory of Planned Behavior. Our primary outcome was the intention to use wiki-based order sets in clinical practice. We also assessed participants’ attitude, perceived behavioral control, and subjective norm to use wiki-based order sets. Paired pre- and post-Likert scores were compared using Wilcoxon signed-rank tests. The post-questionnaire also included open-ended questions concerning participants’ comments about the wiki, which were then classified into themes using an existing taxonomy.

**Results:**

Twenty-eight emergency physicians were enrolled in the study (response rate: 100%). Physicians’ mean intention to use a wiki-based reminder was 5.42 (SD 1.04) before the intervention, and increased to 5.81 (SD 1.25) on a 7-point Likert scale (*P*=.03) after the intervention. Participants’ attitude towards using a wiki-based order set also increased from 5.07 (SD 0.90) to 5.57 (SD 0.88) (*P*=.003). Perceived behavioral control and subjective norm did not change. Easier information sharing was the most frequently positive impact raised. In order of frequency, the three most important facilitators reported were: ease of use, support from colleagues, and promotion by the departmental head. Although participants did not mention any perceived negative impacts, they raised the following barriers in order of frequency: poor organization of information, slow computers, and difficult wiki access.

**Conclusions:**

Emergency physicians’ intention and attitude to use wiki-based order sets increased after having access to and being motivated to use a wiki for 6 months. Future studies need to explore if this increased intention will translate into sustained actual use and improve patient care. Certain barriers need to be addressed before implementing a wiki for use on a larger scale.

## Introduction

Clinical practice does not always reflect best evidence. High proportions of inappropriate care have been reported in different health care systems and settings and have a huge impact on both patient outcomes and health care costs [1]. Information and communication technologies (ICTs), such as computerized decision support systems, have been suggested as a possible solution for improving research uptake and increasing evidence-based practice [2,3]. However, these systems have yet to deliver the expected benefits despite the billions of dollars governments have invested in anticipation of improving care and reducing costs [4]. Moreover, some health care professionals have rejected these ICTs on the grounds that they are slow, incompatible with work processes, difficult to access, costly to implement, and cannot be adapted to local practices [4-8]. Furthermore, local initiatives to adapt the content of various clinical decision support systems seem to be restricted to a small number of hospitals, and tools are mostly designed for local use only [6]. Transfer of these local initiatives to the larger health care community is often slow and complex. Wikis are an open-source and low-cost means of accelerating innovation that could offer a solution to these problems by reducing duplication of effort, optimizing use of existing resources, and by engaging local stakeholders [6,9-12].

Wikis are knowledge management platforms that may empower stakeholders to implement evidence-based decision support tools in different areas of health care [13-15]. A wiki is a website that uses a novel technology to allow people to view and edit website content, with viewing and editing privileges determined by various levels of access. Many health organizations have started using wikis to manage knowledge and coordinate care [10,16,17].

In emergency departments (EDs), where shift work is prevalent, getting health care professionals to collaborate in creating, using, and updating decision support tools is particularly difficult [9,13,18]. EDs often translate clinical practice guidelines into order sets (ie, predefined groupings of standard medical orders for a condition, disease, or procedure) to remind their clinicians about best practice. However, these order sets must be adapted to local practice [19]. A wiki could permit multiple stakeholders in one or many EDs to collaborate asynchronously in the updating and creation of order sets, decreasing duplication, and reducing the time needed [9,11,20]. However, despite increasing evidence supporting the use of wikis in various settings, there is a lack of knowledge about the impact a wiki has on the implementation of best practices. Our overarching research program aims at evaluating the impact of a wiki containing various order sets on the implementation of best practices in trauma care [11,12]. However, before we can achieve this, we must identify the factors influencing professionals’ use of the wiki [9,20,21] to facilitate its implementation. Many factors (eg, openness, instant publication, non-monetary incentives, group affiliation, motivation, strong leadership, active coordination) have been shown to improve contributions to wiki projects in other fields such as education [23,24], sociology [25,26], informatics [27-31], and management [32-34]. However, very few theory-based investigations have been led in the field of health care to understand how health professionals could use wikis’ potential to improve collaborative knowledge implementation in interprofessional settings like EDs [9,22,35,36]. In addition to the importance of studying how to get health professionals to contribute to a wiki, it is also important to know how to get to use and trust collaboratively created content. Our hypothesis is that if we can optimize the clinical use of our wiki by developing a theory-based intervention that will target the behavioral determinants that influence wiki use by health professionals, we will be capable of developing an effective and low-cost intervention that will improve the implementation of best practices in emergency settings. To test our hypothesis, we wanted to assess the impact of actually contributing to a wiki on emergency physicians’ (EPs) intention and behavioral determinants to using the wiki in clinical practice. Thus, our objectives were: (1) to describe the use of a wiki to create structured order sets for a single ED; (2) to evaluate whether the use of this wiki changed emergency physicians’ future intention to use wiki-based order sets; and (3) to understand the impact of using the wiki on the behavioral determinants to use wiki-based order sets.

## Methods

### Setting

As of June 2013, the ED at Hôtel-Dieu de Lévis (HDL), a university-affiliated hospital in Lévis, Quebec, was moving into a newly renovated and much larger Emergency Department (4625 m^2^compared to 1387 m^2^). The ED was also planning an evolution away from paper-use with a new computer-physician order entry (CPOE) system (Med-Urge^TM^, MédiaMed Technologies, Mont-Saint-Hilaire, Québec, Canada). This system allows local order sets to be entered in the system; however, it does not have an open collaborative writing application to help clinicians develop the order sets collaboratively. Thus, the department head in collaboration with our research team decided to use a wiki platform (a Google Sites^TM^ wiki) to create the order sets collaboratively with the 28 EPs in the Emergency Department. This provided the opportunity to assess EPs’ intention to use a wiki-based order set before and after 6 months of actually using the wiki to create and edit the order sets for the new CPOE system.

### Study Design, Participants Recruitment, and Baseline Data Collection

This study is a prospective pre/post-intervention mixed methods study among the EPs at HDL. All EPs at HDL were eligible to participate except the principal author and the departmental head (PB). After receiving ethics committee approval in October 2012, we presented the research project to the EPs at HDL at their monthly departmental meeting. All eligible participants were sent an invitation by email to respond to a previously validated Web-based survey [21]. This questionnaire contained an HTML link to a 6-minute YouTube video [9,21] depicting a physician using a wiki-based order set for the management of severe traumatic brain injury victims in the ED. Participants had to view this video before responding to the questionnaire. The video allowed participants to understand all the small implicit lead-in behaviors necessary to using a wiki-based order set in clinical practice (eg, logging onto the Internet, using a keyboard to type in the search terms to find the wiki-based order set, checking off the appropriate prescriptions suggested by the wiki-based order set). After viewing the video, the questionnaire, based on the Theory of Planned Behavior (TPB) [37], contained 55 items that measured the direct and indirect TPB constructs (intention, attitude, subjective norm, perceived behavior control, behavioral beliefs, control beliefs, and normative beliefs) including 10 sociodemographic items. The TPB is well known for its application to the study of health care professionals’ behaviors [38,39]. Furthermore, the TPB questionnaire used for this survey has been formally validated for the behavior being studied in this study and has adequate test-retest reliability (Multimedia Appendix 1) [21]. Participants who preferred a paper-based questionnaire were sent a paper copy of the survey and an email containing the HTML link to the YouTube video. The video was also presented during the monthly departmental meeting in October 2012. After the initial invitation, participants received two reminders at two-week intervals to respond to the questionnaire between October and November 2012.

### Intervention

The intervention consisted of making the Google Sites wiki available to all 28 EPs at HDL and receiving the instructions from the departmental head to use the wiki to create a series of order sets that would be transferred to the new CPOE system in June 2013. In December of 2012, the department head presented a brief overview of Google Sites’ functionalities to the EPs at the monthly departmental meeting where all EPs were invited to attend. Google Sites was presented as the wiki platform that would be used to create the different order sets for the Department. Google Sites is a structured wiki- and web-creation tool offered by Google as part of the Google Apps for Work productivity suite. Google Sites allows anyone to create a team-oriented website where multiple people can collaborate and share files. We chose Google Sites because it was free (with a maximum of 100 MB of storage), easy to use, and most members of our ED already had Gmail accounts and were accustomed to using Google applications such as Google Docs. The advantages to using Google Sites were that we did not need users to know HTML or any wiki markup language and many different Webpage templates existed. The possibility to manage three different levels of user access was also an interesting feature that influenced our choice to use Google Sites. These three levels of permissions are: Owner, Editor, and Viewer. Owners have full permissions to modify design and content of the entire Google Site, whereas editors cannot change the design of the site. Viewers can only view the site and are not permitted to make any changes to text or otherwise. All the participants in this study were given editor-level access. Additional guidance regarding the steps involved for editing an order set were presented in person and on the home page of the wiki.

A Google Sites wiki page, called “Urgence HDL Informatisation” [40], was created and then presented to the group. This Google Sites wiki was created to contain collaboratively created order sets (written in French) and information related to the Department’s transition towards the new CPOE system. The departmental head then asked all members to design at least one order set and then to review those made by their colleagues. The departmental head asked members to accomplish these tasks as part of their mandatory departmental responsibilities without any additional form of remuneration. An initial list of order set titles was created by the departmental head based on a list of the most frequent ED reasons for consulting the ED (eg, chest pain, gastroenteritis, sepsis). Members of the Department could also add to this list with their own order set topics. After this meeting, the EPs used the wiki to create and edit order sets for 6 months in preparation of their use of the new CPOE in June 2013. From December 2012 to May 2013, the departmental head reminded members at each monthly meeting to complete their wiki-based order set. All ED physicians had editing privileges during the duration of this trial. Although access to reading wiki content was available to anyone with the HTML link, wiki editing and pages revision history was only available to wiki editors. Once all members had completed their order sets, we systematically peer-reviewed them during a Department meeting and further improved them before the department head conducted a final review before exporting them manually into the new CPOE system by copying and pasting content into the new CPOE system. Participants were also encouraged by the departmental head to ask questions and add comments during or after meetings using the discussion thread function linked to each Google Sites page. Although our Google Sites wiki was planned to remain available after the study, it was only created for the purpose of this study and to create the order sets for the new CPOE system.

The purpose of getting emergency physicians to contribute content to this Google Sites wiki was to allow them to get to know how a wiki works and how its content is created collaboratively. As previously explained in the introduction, we wanted to explore the impact of this intervention on emergency physicians’ future intention to use the wiki to inform decision-making in clinical practice. Even though our intervention is focused on “contributing to a wiki”, it is important to understand that our pre and post-questionnaires only focused on participants’ intention “to use a wiki-based order set” in clinical practice.

### Outcomes and Post-Intervention Data Collection

The primary outcome measured following this intervention was the intention to use the wiki-based order set in a clinical context. Other secondary outcomes were also measured using our TPB questionnaire: attitude, perceived behavioral control, subjective norm, and the indirect TPB constructs (behavioral beliefs, control beliefs, and normative beliefs). We also used Google Analytics^TM^ to collect daily wiki usage statistics. These statistics were only available after the wiki was launched. The post-intervention questionnaire included open-ended questions to better understand participant’s positive and negative views about using the wiki. We also asked participants to make suggestions to improve the wiki. Again, both Web-based and paper-based versions were available, but participants were not asked to view the video prior to completing the post-intervention questionnaire.

### Data Analysis

We imported data from completed Survey Monkey questionnaires into an Excel spread sheet as well as daily usage statistics from Google Analytics. We compared the means, medians, interquartile ranges, and confidence intervals for each pre- and post- intervention question. Considering our small sample size, we conducted Wilcoxon signed-rank tests for continuous variables. We conducted a post-hoc analysis to compare the demographic characteristics and the measured behavioral determinants of participants who reported using the wiki during the 6-months study. For any missing data for single questionnaire items, we imputed the average of the other items measuring the same construct only if data was available from a minimum of two other items. A biostatistician performed the statistical analyses using SAS version 9.3 (SAS Institute Inc, Cary, NC, USA).

Two research assistants independently analyzed answers to open-ended questions by classifying the content by theme using two previously developed taxonomies (ie, positive/negative impacts, barriers/facilitators) [13]. They classified answers as a perceived positive and/or negative impact when a participant claimed that using the wiki had an impact on a clinical process or a clinical outcome. They classified answers as barriers/facilitators when the idea expressed by the participant was a barrier or facilitator to using the wiki. They then classified the comments in each theme by frequency of reporting.

## Results

### Participant Characteristics

Twenty-eight EPs from HDL completed both questionnaires, for a response rate of 100%. As seen in Table 1 , most participants were mid-career, male physicians with emergency medicine certification issued by the College of Family Physicians of Canada. Collaborative writing applications reported as having been previously used for personal reasons were: Wikipedia and Google Docs. One physician reported having previously edited a document using a collaborative writing application, specifically Google Docs. Other tools that clinicians mentioned as being used frequently were Dropbox and Evernote.

**Table 1:** Characteristics of participating emergency physicians (EPs).

Variables		EPs (n=28)
**Age (years)**		
	Mean (SD)	40.7 (7.4)
	Median (IQR)	41 (35-47)
**Gender, n (%)**		
	Female	7 (25%)
	Male	21 (75%)
**Emergency medicine certification, n (%)**		
	Royal College of Physicians and Surgeons of Canada	7 (25%)
	College of Family Physicians of Canada	21 (75%)
Previous professional use of a wiki, n (%)		7 (25%)
Previous personal use of a wiki, n (%)		19 (68%)
Previous editing of a wiki, n (%)		1 (3.5%)

### Order Sets Developed

During the 6-month study, the wiki was used to create 68 order sets for a variety of conditions seen in the ED in the fields of anesthesia and critical care (n=9), neurology (n=9), gynecology-obstetrics (n=6), psychiatry (n=6), cardiology (n=5), pediatrics (n=5), trauma (n=4), rheumatology (n=4), ophthalmology/otorhinolaryngology (n=4), infectious diseases (n=4), gastroenterology (n=3), geriatrics (n=3), respirology (n=2), orthopedics (n=2), and hematology/oncology (n=2). A complete list of these order sets is available from the wiki itself [40]. In all, 15/28 (54%) participants created at least one order set and 13/28 (46%) did not create any (median of 0.5 order set per participant). The three most productive participants created more than 15 order sets each.

### Post-Intervention Intention to Use Wiki-Based Order Sets

After 6 months of using the wiki to create order sets, participants were asked to respond to the post-intervention questionnaire on May 12, 2013 and the last participant responded on May 30, 2013. EPs’ intentions to use a wiki-based order set to promote best practices in EDs increased from 5.42 to 5.81 on the Likert scale, representing a 0.39 point increase (*P*=.03) (Table 2). This difference in mean Likert scores is likely not to be clinically significant being that it is below the threshold of a 0.6 point increase (half of the standard deviation in our sample). Among all the other direct and indirect TPB constructs, we also found that attitude and normative beliefs increased after the intervention. Finally, none of the constructs were negatively influenced by our intervention.

**Table 2:** Pre- versus post- intervention measurement of Theory of Planned Behavior constructs (measured on a 7-point Likert scale).

Constructs		Pre-intervention			Post-intervention		*P*
	Mean (SD)		Median	Mean (SD)		Median	
Intention	5.42 (1.04)		5.33	5.81 (1.25)		6.00	.03
Perceived behavioral control	5.46 (1.06)		5.67	5.80 (1.28)		6.00	.12
Subjective norm	4.21 (1.28)		4.33	4.58 (0.93)		4.67	.08
Attitude	5.07 (0.90)		5.00	5.57 (0.88)		5.75	.003
Normative beliefs	5.17 (0.96)		5.18	5.74 (0.75)		5.86	<.001
Control beliefs (facilitators)	6.49 (0.63)		6.64	6.68 (0.36)		6.82	.17
Control beliefs (barriers)	3.77 (1.33)		3.90	3.95 (1.18)		4.10	.40
Behavioral beliefs	6.00 (0.73)		6.13	6.20 (0.66)		6.31	.13

### Data on Use of the Wiki

**Figure 1:**
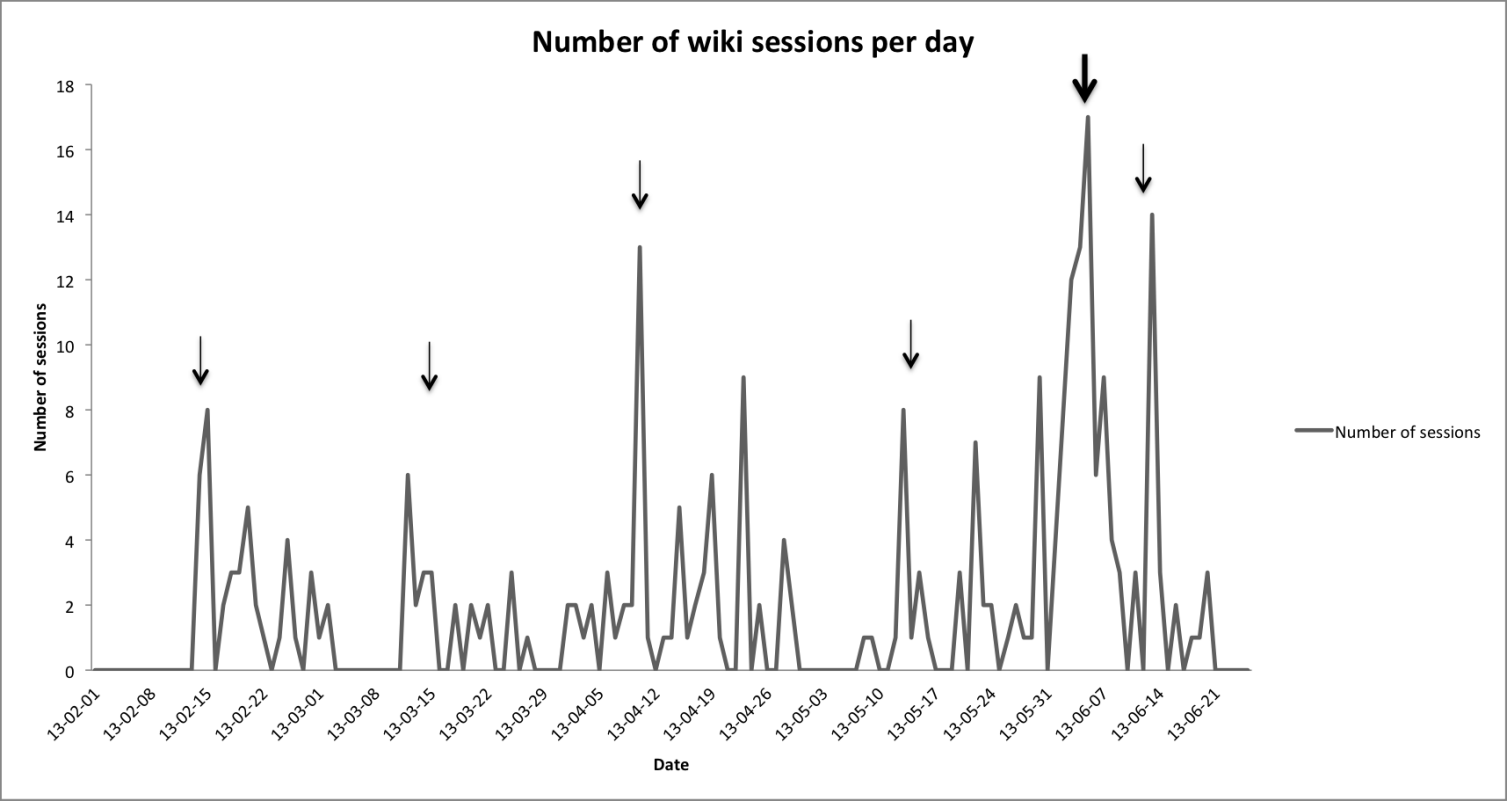
Number of wiki sessions per day between February 1 and June 25, 2013 (narrow arrows: monthly departmental meetings; bold arrow: simulation in new ED).

Once all participants had replied to our pre-intervention survey in November 2012, participants were given access to use the wiki starting in December 2012. The last participant receiving access to the wiki was on January 14, 2013. It was not until February 2013 that we created a Google Analytics account to monitor its ongoing use. With the usage data available (Figure 1), we observe that more sustained use began between February 2 to March 2. Then, its use increased over the period of March 12 to 19. There was a further increase of visits between the 30^th^May and the 9^th^of June. Monthly departmental meetings were held on February 13, March 13, April 10, May 15, and June 12 (indicated by narrow arrows on Figure 1) during which the departmental head reminded the participants to contribute to the wiki. On June 5, the wiki experienced its greatest use with 17 visits. On this day, a simulation was held to practice using the new ED infrastructures including the new CPOE system (bold arrow in Figure 1). The new ED officially opened on June 17, 2013.

On average, the wiki was used 1.9 times per day by 54% (15/28) of the participants. Although there was a trend for non-users to be older, male, specialized EPs, there were no statistically significant differences between wiki users and non-users (Multimedia Appendix 2). Six participants used the discussion thread function to add comments to different wiki pages. This included one participant who made comments on 46 different order sets. The content of these comments concerned dosing of medications, suggestions to improve the order sets and ideas for other order sets.

### Qualitative Comments About the Wiki

The most frequently mentioned positive perceived impact was that the wiki facilitated information and knowledge sharing (n=3) (Table 3). There were no negative perceived impacts mentioned. However, many different barriers were mentioned (Table 4). The top three barriers reported were: the organization of information needed to improve (n=7), the computers used were slow (n=6), and that access to the wiki was difficult (n=5). Even though restricted access was mentioned as a barrier to using our wiki, 4 participants also mentioned the opposite view that having an open access wiki would be a potential barrier for future clinical use. Although the wiki was consulted 23 times using an iPad, 16 times by an iPhone, and once by a Motorola XT720 MOTOROI device, there were no comments about difficult access using mobile devices. The most frequently reported facilitators to using the wiki were the wiki’s ease of use (n=5), the support and promotion by colleagues (n=3), and also the administrative support (n=2) (Table 5).

**Table 3:** Perceived positive impacts about using the wiki-based order sets.

Perceived positive impact	n^a^
Information and knowledge sharing	3
Feedback (eg, “enables feedback from my colleagues”)	3
Standardization of practices	2
Better access to information	1

^a^n=the number of single mentions by participants of each positive impact. Nine participants made comments about the wiki’s perceived positive impact on their online survey.

**Table 4:** Barriers to using the wiki-based order sets.

Barriers	n^a^
Organization of information (eg, “layout and visual presentation”)	7
Material resources - Slow speed of computers	6
Material resources (access to wiki)	5
Open access wiki (eg, “possibility that anyone can modify content”)	4
Lack of webmetric tool to present recent changes	1
Time constraints to edit	1
Lack of familiarity with the wiki (ie, need to learn how to use the platform)	1

^a^n=the number of single mentions by participants of each barrier. Eight participants made comments about barriers to wiki use in their online survey.

**Table 5:** Facilitators to using the wiki-based order sets.

Facilitators	n^a^
Ease of use	5
Support and promotion by colleagues	3
Administrative/organizational support (eg, “department head”)	2
Motivation to contribute consistent with clinical needs	1
Awareness of the existence of the wiki	1
Triability (eg, “trying the platform alone”)	1
Easy access	1
Incentives (eg, “use made mandatory”)^a^	1
Appearance of wiki	1

^a^This facilitator was not described in the taxonomy used; n=the number of single mentions by participants of each facilitator. Eleven participants made comments about facilitators for wiki use in their online survey.

## Discussion

### Principal Findings

Using a previously validated theory-based questionnaire, we determined that using a wiki to construct a series of order sets during a 6-month period increased the intention of using such a Web-based tool. Moreover, we also demonstrated that a wiki could be used to construct order sets in a single Emergency Department with 54% of our participants contributing at least one order set to the wiki during our study. Intention among EPs to use a wiki-based reminder increased by 0.39 points on a 7-point Likert scale (*P*=.03) after having access to the wiki for a period of 6 months. This increase was not clinically significant based on our cut-off for clinical significance (<0.6 points on our 7-point Likert scale) [41]. For the other TPB constructs, our intervention increased the attitude towards using a wiki-based order set to promote best practices. Interestingly, all these increases occurred even if the initial levels of intention and attitude were high.

We also identified specific aspects of the complex task of accessing and using a wiki that inform on improvements to be made and appreciated qualities to be maintained. For example, access to editing wiki content needs be controlled, but this needs to be balanced with better access to viewing wiki content (ie, better bedside access). Layout of information and computer performance need to be improved. Having the support and leadership from the departmental head was noted as an important facilitator for any future implementation. This support was instrumental to manage our wiki platform as our departmental head used monthly departmental meetings to stimulate collaborative writing periods among ED members with varying levels of comfort with wikis and technology in general. In this respect, the Google Sites wiki was perceived as easy to use, easy to access (eg, using mobile devices) and triable. These results lead us to the following observations.

To our knowledge, this study is the first to evaluate the effect of using a wiki on EPs’ behavioral determinants of using wiki-based order sets to promote best practices. Other authors have used the Technology Assessment Model to explore how health professionals use and contribute to social media in general to share medical knowledge with other physicians in the medical community at one point in time [22]. Kohli et al [42] evaluated the use of a wiki for document sharing among residents in radiology and their contribution to updating and editing the wiki, but did not use any theoretical framework to assess the impact of this intervention on behavioral determinants. In contrast, our study measured the change in intention over time and provided an understanding about how our intervention acted on the behavioral determinants.

Although our intervention was not specifically designed to address any of the theoretical social cognitive determinants of the TPB, it did have a positive impact on three of these determinants (intention, attitude, and normative beliefs). Using intervention mapping [43] or the Theoretical Domains Framework [44,45], future theory-based interventions could be built to specifically address certain cognitive determinants.

In particular, social norm and control beliefs were the lowest among the TPB constructs and we would need to specifically design an intervention targeting these constructs in a larger study. Even though the social norm construct did not increase significantly, the normative beliefs construct did increase significantly, meaning that our intervention had some social effect. By acting with the support of the departmental head and asking different colleagues to support each other and collaborate in creating their wiki order sets, we possibly unwillingly acted on certain normative beliefs. Surprisingly, none of the qualitative comments analyzed mentioned a negative referent that could be a target for a future intervention.

Although our participants’ high previous personal and professional use of wikis did not seem to influence their 6-month use of our wiki, other authors have previously shown the importance of past behavior/habit in predicting behavior among health professionals [46,47]. Habit creation supported by reminders to use the wiki at our monthly departmental meetings likely increased reported 6-month use and future intention to use the wiki. This also resulted in a relatively high contribution rate with 54% of our participants contributing at least one order set to the wiki during our study. Although this contribution rate was unequally distributed among our participants (with some participants contributing many order sets and others not contributing any), this contribution rate is higher than contribution rates (3-22%) reported in other studies [36,48] and represents an increase compared to participants’ self-reported baseline contribution rate prior to starting this study (3.5%). The mere measurement effect must also be considered as a potential explanation for the increase in intention, use, and contribution rate [49].

### Limitations

Our study has some limitations. First, our sample of EPs at HDL may not represent the beliefs of EPs elsewhere. In particular, EPs in our sample reported lower prior wiki use for professional purposes than reported in a recent scoping review of wiki use in health care [13]. This review identified studies reporting a range of usage rates ranging from 55% for consultants and 80% for junior physicians [48,50]. Moreover, the social cognitive determinants of our study population may have been influenced by the fact that the study was being carried out in their hospital as well as by their proximity to the research physicians carrying out the study. The EPs were also aware that the ED was evolving away from paper use and were therefore possibly more inclined to use a wiki than physicians working in a paper-based center.

Second, we did not adjust our significance level for multiple comparisons. Rothman argues that not making adjustments for multiple comparisons leads to fewer errors of interpretation when the data under evaluation are not random numbers but actual observations on nature [51]. Furthermore, scientists should not be reluctant to explore leads that may turn out to be wrong because they might miss possibly important findings [52]. For this reason, further studies will still be needed to confirm our findings.

Third, our use of the TPB limits our capability to directly assess the importance of environmental factors such as organizational readiness for change. The use of the Theoretical Domains Framework to inform our theory-based intervention could correct this [53,54].

Fourth, our questionnaire did not measure the determinants of contributing to the wiki, in addition to consulting it. By definition, a wiki is a product of its users and remains relevant only if its users continue to update it and create new content. Getting experts and other members of a wide community to contribute to a collaborative writing project is a difficult task and a theory-based approach will be needed to stimulate and promote this behavior [18,36,55]. Several further behaviors will need to be studied in the future, but we chose the one we felt to be the most important (using the wiki).

Fifth, our study did not evaluate the quality of the order sets created. Although all order sets were peer-reviewed by our departmental head and reviewed by all participants during our monthly departmental meetings, future studies will need to explore how to measure the quality of order sets and how a wiki collaborative writing platform can contribute to improving the quality of order sets currently in clinical use.

Finally, our use of Google Sites will potentially limit the future expansion of our wiki content and its integration into other health information technology to support clinical decision-making. Even though our content is free and open-source, the platform itself is not open-source meaning we cannot modify the wiki programming to integrate it directly into our CPOE, which was not an open-source program either. Therefore, there still remains a gap between the collaboratively created order sets in our wiki and their actual clinical use. Future explorations of completely open-source solutions and open-source CPOE could help solve this problem [11,56].

### Future Studies

Future studies will also have to try to determine what represents a clinically significant increase of intention to use a wiki-based reminder. Moreover, rigorously designed implementation studies with larger samples are needed to determine the impact of wiki-use in trauma care. Better understanding of the impact of editing and using a wiki on the behavioral determinants for future wiki-use will also be important to explore in order to develop a sustainable and scalable knowledge translation intervention. Future studies also need to investigate how a collaborative writing platform can be used to produce high-quality evidence-based order sets and better integrate these collaboratively created order sets into CPOE systems that are more responsive and adaptive to local clinical needs.

### Conclusion

Using wiki-based order sets in trauma care for the promotion of best practices seems possible given that EPs’ intentions increased through its use. However, the clinical impact of this novel intervention remains to be verified using a rigorous study design with a larger population. Further development of our wiki will also need to consider the different barriers and facilitators identified by our users to build a highly usable and reliable evidence-based clinical resource.

## Appendix

Multimedia Appendix 1 Theory of planned behavior questionnaire.

Multimedia Appendix 2 Comparison of wiki users and non-users at 6 months.

## Abbreviations

CPOE: computer-physician order entry
ED: emergency department
EP: emergency physician
HDL: Hôtel-Dieu de Lévis
HTML: Hyper Text Markup Language
ICT: information and communication technologies
TPB: Theory of Planned Behavior
