# Supplementary material for: Impact of Implementing a Wiki to Develop Structured Electronic Order Sets on Physicians' Intention to Use Wiki-Based Order Sets
Source: JMIR Med Inform. 2016 May 17;4(2):e18. doi: 10.2196/medinform.4852 (PMC4909394; doi:10.2196/medinform.4852)
Supplement: Multimedia Appendix 1 [file medinform_v4i2e18_app1.pdf]

STUDY ON WIKI USE

PHYSICIAN QUESTIONNAIRE

WIKI PROJECT (A)

Date: ________________
dd/mm/yyyy

1. This questionnaire is about the use of wiki-based reminders that promote best practice in the management of severe cranial trauma in emergency rooms in the province of Quebec.
2. Although some of the questions may seem repetitive, it is very important that you answer them all anyway.
3. Your choice of answers should best reflect your opinion or your situation.
4. There are no right and wrong answers.
5. Your answers will remain confidential.
6. The questionnaire should take about 10 minutes.
Your participation in this survey is voluntary and your data will remain confidential. Returning the completed questionnaire will be considered an expression of your consent to participate in the project.
For any comments or questions about this research project please contact Susie Gagnon, Research Professional, at 418-835-7121 ext. 6267.

Consent:

I understand that the data collected by this questionnaire will be anonymous. I understand that it will be treated with confidentiality and in no circumstances will be associated with my institution when the results are made public. I have noted the above information and I freely consent to participate in this study. I understand that I may stop answering the questionnaire at any moment without prejudice.

- [ ] Yes
- [ ] No

Before you begin, please watch this video.

Definition of a wiki:

- A wiki is a website programmed to allow anyone who has access to it to edit (change) its content. It is thus a collaborative work tool, and could contain treatment protocols, for example, or decision algorithms. In the healthcare context, a wiki could enable doctors and other professionals to share, update and edit reminders based on the most recent evidence. Healthcare professionals who had access to a wiki in their hospital could consult it directly in their workplace.
- For example, in the case of a severe cranial trauma, a wiki-based reminder could be brought up online on a computer and the team could use the information in their response.

This questionnaire seeks your opinion on the possible use of a wiki-based reminder that promotes best practices in managing severe cranial trauma in emergency rooms in the province of Quebec.

1. **I would feel capable of using a wiki-based reminder** that promotes best practice for managing severe cranial trauma in emergency rooms in the province of Quebec.

1	2	3	4	5	6	7

Strongly disagree **Strongly agree**

2. **If I wanted to, I feel confident that I could use a wiki-based reminder** that promotes best practice for managing severe cranial trauma in emergency rooms in the province of Quebec.

1	2	3	4	5	6	7

Strongly disagree **Strongly agree**

3. **I feel social pressure to use a wiki-based reminder** that promotes best practice for managing severe cranial trauma in emergency rooms in the province of Quebec.

1	2	3	4	5	6	7

Strongly disagree **Strongly agree**

4. **For me to use a wiki-based reminder** that promotes best practice for managing severe cranial trauma in emergency rooms in the province of Quebec would be:

1	2	3	4	5	6	7

Very difficult **Very easy**

5. **I am expected to use a wiki-based reminder** that promotes best practice for managing severe cranial trauma in emergency rooms in the province of Quebec.

1	2	3	4	5	6	7

Strongly disagree **Strongly agree**

6. **I intend to use a wiki-based reminder** that promotes best practice for managing severe cranial trauma in emergency rooms in the province of Quebec.

1	2	3	4	5	6	7

Strongly disagree **Strongly agree**

7. **The people who are most important to me think I should use a wiki-based reminder** that promotes best practice for managing severe cranial trauma in emergency rooms in the province of Quebec.

1	2	3	4	5	6	7
**Strongly disagree**						**Strongly agree**

8. **I estimate the odds of my using a wiki-based reminder** that promotes best practice for managing severe cranial trauma in emergency rooms in the province of Quebec to be:

1	2	3	4	5	6	7
**Very low**						**Very high**

9. **I will use a wiki-based reminder** that promotes best practice for managing severe cranial trauma in emergency rooms in the province of Quebec.

1	2	3	4	5	6	7
**Very unlikely**						**Very likely**

10. **For me, using a wiki-based reminder** that promotes best practice for managing severe cranial trauma in emergency rooms in the province of Quebec would be:

(*tick* ***one box*** *in* ***each*** *of these three scales***)**

1	2	3	4	5	6	7
**Very unpleasant**						**Very pleasant**

1	2	3	4	5	6	7
**Totally useless**						**Very useful**

1	2	3	4	5	6	7
**Very unsatisfying**						**Very satisfying**

Please use this scale to answer the question that follows.

1	2	3	4	5	6	7
**Strongly disagree**						**Strongly agree**

11. The following people would **approve** of **my use of a wiki-based reminder** that promotes best practice for managing severe cranial trauma in emergency rooms in the province of Quebec:

The **nurses** at my hospital

1 2 3 4 5 6 7

The **doctors** at my hospital

1 2 3 4 5 6 7

The hospital staff in regional/remote hospitals who are less exposed to severe cranial trauma

1 2 3 4 5 6 7

The **younger generation of staff** at my hospital

1 2 3 4 5 6 7

The **respiratory therapists** at my hospital

1 2 3 4 5 6 7

The **trauma team** at my hospital

1 2 3 4 5 6 7

The **administration** at my hospital

1 2 3 4 5 6 7

My **patients**

1 2 3 4 5 6 7

The **specialists (intensive care, surgeons)** at my hospital

1 2 3 4 5 6 7

Please use this scale to answer the question that follows.

1	2	3	4	5	6	7
Strongly disagree						Strongly agree

12. The following people would **disapprove** of **my use of a wiki-based reminder** that promotes best practice for managing severe cranial trauma in emergency rooms in the province of Quebec would be disapproved of by

People who are **opposed to the standardized care** offered at my hospital

**1**	**2**	**3**	**4**	**5**	**6**	**7**

People at my hospital **who are not used to computers**

**1**	**2**	**3**	**4**	**5**	**6**	**7**

13. **I would feel capable of using a wiki-based reminder** that promotes best practice for managing severe cranial trauma in emergency rooms in the province of Quebec:
[*Tick **one box** for **each** of the 9 statements below*]

	Strongly disagree	More or less disagree	Slightly disagree	Neither agree or disagree	Slightly agree	More or less agree	Strongly agree
a) **if** it was simple to use.							
b) **if** a computer was available at the patient's bedside							
c) **if** the information had been validated by a peer review committee.							
d) **if** access to the reminder was quick.							
e) **if** the institutional did not control access (e.g. passwords, blocked sites).							
f) **if** it was properly integrated into my work activities.							
g) **if** it was accessible via my mobile device (e.g. iPod, iPhone, iPad, Blackberry).							
j) **if** my hospital had internet access.							
k) **if** the site was well designed.							

14. **I would feel capable of using a wiki-based reminder** that promotes best practice for managing severe cranial trauma in emergency rooms in the province of Quebec:

[*Tick **one box** for **each** of the 5 statements below*]

	**Strongly disagree**	**More or less disagree**	**Slightly disagree**	**Neither agree or disagree**	**Slightly agree**	**More or less agree**	**Strongly agree**
a) **even if** the information wasn't regularly updated.							
b) **even if** I had very little time.							
c) **even if** the information changed often.							
d) **even if** the authors were not identified.							
e) **even if** I didn't know who was legally responsible.							

15. **If I used a wiki-based reminder** that promotes best practice for managing severe cranial trauma in emergency rooms in the province of Quebec, it would:
[*Tick* ***one box*** *for* ***each*** *of the 8 statements below*]

	Strongly disagree	More or less disagree	Slightly disagree	Neither agree or disagree	Slightly agree	More or less agree	Strongly agree
a) enable me to refresh my memory.							
b) give me access to evidence-based information.							
c) enable sharing of information with other hospitals.							
d) standardize practices.							
e) centralize information and protocols.							
f) reduce intervention errors.							
g) give me access to expert opinion.							
h) reduce my stress level.							

Sociodemographic information

16. How old are you? ______

17. What sex are you?
F ☐ M ☐

18. Do you have a certificate in emergency medicine?
Yes ☐→ ☐ College of Family Physicians of Canada
☐ Royal College of Physicians and Surgeons of _______

No ☐→ My specialty is ____________________________

19. What hospital do you work in? ________________________

20. How many years have you worked in emergency (since your residency)? _____

21. Is there a computer with internet access in your emergency room?
Yes ☐ No ☐

22. Do you currently use a wiki in your professional life (e.g. Wikipedia)
☐ Yes → Which one? _______________How often?_____________
☐ No

23. Do you currently use a wiki in your personal life?
☐ Yes → Which one? _______________How often? _____________
☐ No

24. Have you ever edited a wiki (made changes to the website)?
☐ Yes → Which one? _______________ How often? _____________
☐ No

25. Are you a member of a traumatology committee (local or regional)?
☐ Yes
☐ No

The questionnaire is over!

Please make sure you have answered all the questions.

THANK YOU FOR YOUR COLLABORATION

If you have any comments or suggestions regarding this questionnaire or this study please write them here:
