# Supplementary material for: Impact of Implementing a Wiki to Develop Structured Electronic Order Sets on Physicians' Intention to Use Wiki-Based Order Sets
Source: JMIR Med Inform. 2016 May 17;4(2):e18. doi: 10.2196/medinform.4852 (PMC4909394; doi:10.2196/medinform.4852)
Supplement: Multimedia Appendix 2 [file medinform_v4i2e18_app2.pdf]

**Multimedia Appendix 2. Comparison of wiki users and non-users at 6 months.**

| Comparison variable | Self-reported wiki use after 6 months | | *P* [a] |
|---|---|---|---|
| | Yes (n=15) | No (n=13) | |
| **Gender** | | | .40 |
| Male, n (%) | 10 (66.7) | 11 (84.6) | |
| Female, n (%) | 5 (33.3) | 2 (15.4) | |
| **Certification** | | | .20 |
| College of Family Physicians of Canada (n (%)) | 13 (86.7) | 8 (61.5) | |
| Royal College of Physicians and Surgeons of Canada (n (%)) | 2 (13.3) | 5 (38.5) | |
| **Previous wiki use** | | | |
| Professional use, n (%) | 5 (33.3) | 2 (15.4) | .40 |
| Personal use, n (%) | 11 (73.3) | 8 (61.5) | .69 |
| **Previous editing of a wiki** | 1 (6.7) | 0 | 1.00 |
| **Age, mean (SD)** | 40.1 (6.9) | 41.3 (8.1) | .68 |
| **Experience level, mean (SD)** | 12.2 (7.2) | 9.5 (6.7) | .33 |
| **Post-intervention TPB[b] constructs** (mean [c] (95%CI)) | | | |
| Intention | 6.13 (1.06) | 5.43 (1.38) | .10 |
| Attitude | 5.73 (0.95) | 5.38 (0.78) | .28 |
| Perceived behavioral control | 5.76 (1.29) | 5.85 (1.32) | .65 |
| Subjective norm | 4.49 (1.01) | 4.69 (0.87) | .55 |
| Behavioral beliefs | 6.19 (0.71) | 6.21 (0.63) | .95 |
| Normative beliefs | 5.57 (0.94) | 5.94 (0.47) | .57 |
| Barriers | 4.10 (1.18) | 3.77 (1.21) | .48 |
| Facilitators | 6.57 (0.43) | 6.80 (0.22) | .22 |

[a] Fisher exact test or Wilcoxon signed-rank test

[b] TPB: Theory of Planned Behavior

[c] Mean Likert scores (7-point scale)
